# Complex Bone Tumors of the Trunk—The Role of 3D Printing and Navigation in Tumor Orthopedics: A Case Series and Review of the Literature

**DOI:** 10.3390/jpm11060517

**Published:** 2021-06-04

**Authors:** Martin Schulze, Georg Gosheger, Sebastian Bockholt, Marieke De Vaal, Tymo Budny, Max Tönnemann, Jan Pützler, Albert Schulze Bövingloh, Robert Rischen, Vincent Hofbauer, Timo Lübben, Niklas Deventer, Helmut Ahrens

**Affiliations:** 1 Department of Orthopedics and Tumor Orthopedics, Muenster University Hospital, Albert-Schweitzer-Campus 1, 48149 Münster, Germany; georg.gosheger@ukmuenster.de (G.G.); sebastian.bockholt@ukmuenster.de (S.B.); mariekemathilda.devaal@ukmuenster.de (M.D.V.); Tymoteusz.budny@ukmuenster.de (T.B.); m.toennemann@uni-muenster.de (M.T.); jan.puetzler@uni-muenster.de (J.P.); albert.schulzeboevingloh@ukmuenster.de (A.S.B.); Vincent.hofbauer@ukmuenster.de (V.H.); dr.luebben@gmail.com (T.L.); niklas.deventer@ukmuenster.de (N.D.); helmut.ahrens@ukmuenster.de (H.A.); 2 Clinic for Radiology, Muenster University Hospital, Albert-Schweitzer-Campus 1, 48149 Muenster, Germany; robert.rischen@ukmuenster.de

**Keywords:** 3D printing, navigation-assisted surgery, tumor orthopedics, oncologic orthopedics, patient specific, tumor surgery, bone defects

## Abstract

The combination of 3D printing and navigation promises improvements in surgical procedures and outcomes for complex bone tumor resection of the trunk, but its features have rarely been described in the literature. Five patients with trunk tumors were surgically treated in our institution using a combination of 3D printing and navigation. The main process includes segmentation, virtual modeling and build preparation, as well as quality assessment. Tumor resection was performed with navigated instruments. Preoperative planning supported clear margin multiplanar resections with intraoperatively adaptable real-time visualization of navigated instruments. The follow-up ranged from 2–15 months with a good functional result. The present results and the review of the current literature reflect the trend and the diverse applications of 3D printing in the medical field. 3D printing at hospital sites is often not standardized, but regulatory aspects may serve as disincentives. However, 3D printing has an increasing impact on precision medicine, and we are convinced that our process represents a valuable contribution in the context of patient-centered individual care.

## 1. Introduction

Bone tumors are rare and account for less than 0.2% of primary malignant neoplasms registered in the database for the European Cancer Registry-based study on the survival and care of cancer patients (EUROCARE) [1].

The classification system for surgical treatment of malignant bone and soft tissue tumors was defined by Enneking [2]. The main objective of surgical treatment is wide tumor resection with sufficient safety margins free of tumor cells [3]. Resection is followed by reconstruction in a subsequent procedure. Tumors close to the trunk, e.g., in the thorax/spine, sacrum and pelvis, involve highly challenging surgical resection techniques compared to tumors of the extremities. Critical aspects are acceptable postoperative function, the preservation of critical neurovascular structures and low perioperative morbidity, mortality and recurrence rates [4,5].

For pelvic tumors, resection defects are classified according to the Dunham system (P1–P4). Functional limitations due to the loss of at least one hip joint component (P2) in combination with very limited reconstruction possibilities make convalescence and mobilization of patients more difficult [6,7,8].

To improve the surgical procedure and outcome, two approaches may be considered. First, navigation-assisted surgical oncology has already proven its potential to reduce operation time, blood loss and the risk of intralesional resections in pelvic and sacral tumors [5,9]. Second, additive manufacturing (AM) processes are advancing in everyday clinical practice and are increasingly being used in trauma surgery, revision endoprosthetics and tumor orthopedics. In tumor orthopedics, in addition to the application of cutting guides for resection, reconstruction using individual implants is particularly notable [10,11,12,13,14,15].

The first and indispensable steps are the preparation of cross-sectional images and 3D modeling. Often, there is special interest in the early initiation of therapy, especially if the tumor progresses rapidly. Depending on the further processing of the 3D model, subsequent work steps may represent limiting factors to the aforementioned model.

By combining established intraoperative navigation with preoperative planning and creation of a patient-specific, full-size 3D-printed model, resection planes can be planned more precisely, healthy tissue and bone substances can be spared, biomechanics can be preserved and, if necessary, findings conventionally classified as inoperable can be surgically treated. Since there is no time required for design and production, therapy can be started almost immediately.

The aims of the present manuscript are to describe the combination of navigation and AM based on a retrospective analysis of a series of five cases and to review the current application of AM in tumor orthopedics.

Achievable advantages of combining navigation and AM include preservation of functional structures and joints, reduction of the risk of injury to internal organs, and planning of the operation using models true to the original to determine the most reasonable alternative for the patient without significant delay. A particular advantage is the opportunity to simulate functions and reconstruction of joint partners in advance in the 3D-printed model.

## 2. Materials and Methods

Five patients (aged 22 to 63 years) with malignancies close to the trunk were surgically treated in our institution between August 2019 and September 2020 using the combination of 3D printing and navigation. Admission of the patients to the combination of 3D printing and navigation was decided by the senior surgeons. In particular, the decision was based on the extent of the tumor and the expected complexity of the procedure, as well as potentially compromised anatomical structures in close spatial relationship to the tumor. Since this is of special relevance for tumors in the trunk region, different clinical entities were grouped as tumors close to the trunk for the present case series. All cases were histopathologically confirmed, staged, and presented to our interdisciplinary tumor board prior to surgery. All patients provided written informed consent for 3D print-assisted planning and navigation. Ethics committee permission was obtained for the present study (Ethics Committee of Medical Association of Westphalia-Lippe and the Medical Faculty of WWU Münster protocol code 2020-282-f-S, date of approval 30 April 2020).

**Figure 1:**
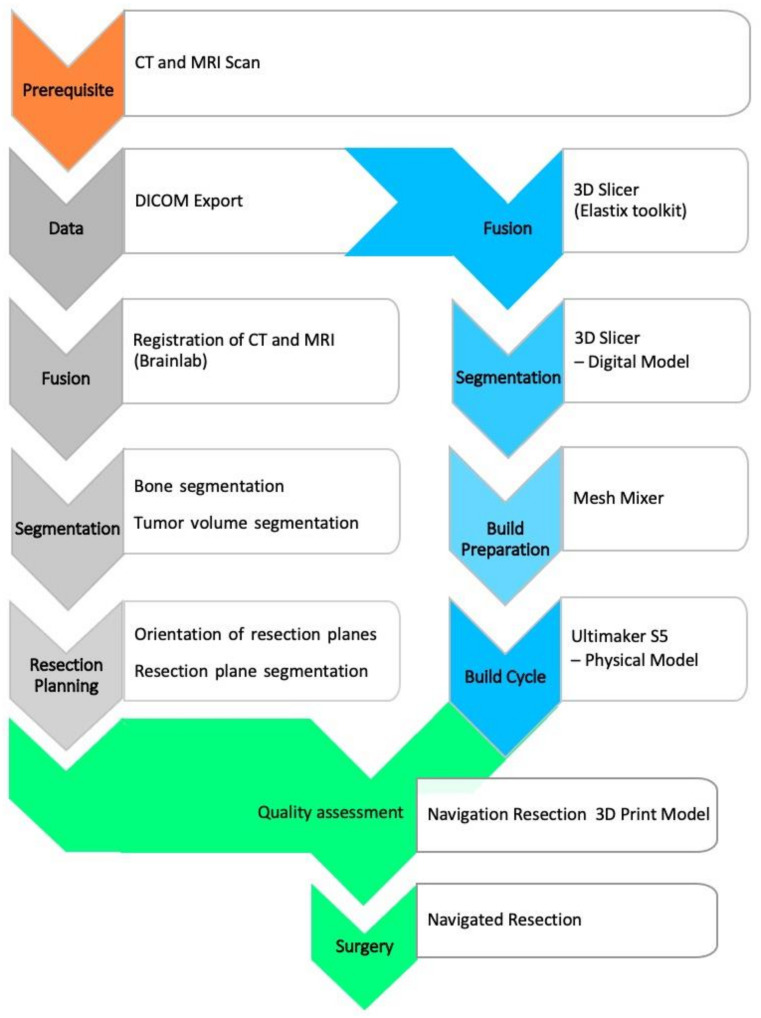
Flow chart for model processing.

Preoperative planning was based on diagnostic cross-sectional imaging. This requires both computed tomography (CT) with a maximum slice thickness of 1 mm [16] and contrast-enhanced magnetic resonance (MR) tomography with 1.5-Tesla field strength and multiaxial reconstruction in the T1/T2 sequences. The subsequent main process includes segmentation, virtual modeling and build preparation, as well as quality assessment, as depicted in Figure 1.

The digital imaging and communications in medicine (DICOM) image data are transferred to software for further processing, segmentation, and model creation. Commercial software is available (for example, the approved medical device from *Materialise;* Leuven, Belgium). However, not every clinic has the resources to use this software, so experimental alternatives are being considered. One of these alternatives is the free open-source software *3D-Slicer* (release 4.10.2 from www.slicer.org (accessed on 27 October 2020)), which has been an established imaging tool in the biomedical sciences [17].

Segmentation is an essential part of model creation. Segmentation is the process of marking image elements layer by layer (2D) in a data set to allow grouping of similar elements into regions or volumes (3D). The tools used were thresholding, region growing, and manual sculpting. CT data can be used to mark anatomical bony structures particularly well, allowing a semi-automated approach. Soft tissue structures may be better segmented using magnetic resonance imaging (MRI) data. Databases of anatomical atlases are steadily improving and increasingly facilitate detection and segmentation [18,19,20]. However, tumors represent a special challenge, as they may contain both bony and soft tissue components and are individually distinct in terms of their geometry and spatial extent. We determined the tumor margin by looking at the transition of the marrow signal from abnormal to normal in T1-weighted MR images [21] (p. 2534).

The 3D model was exported as a mesh surface from planar triangles in a special format as standard tessellation language (STL) and prepared for 3D printing. A wide variety of processes can be suitable for model printing. Essential criteria are sufficient build space, speed, cost, and resolution of the print. We used an *Ultimaker-S5* printer (Ultimaker B.V., Utrecht, The Netherlands) with two print heads for printing with polylactide acid (PLA) polymer. This allows separate printing of support structures or the tumor component for visualization. The free software *Meshmixer* (Autodesk Inc., Dublin, Ireland) was used for preparation. Among other things, it can be used to optimize support structures. Furthermore, the software provides an inspection and repair tool to verify the mesh integrity and avoid print errors. Since the printing process can be fully simulated, material- and time-oriented optimization is possible. Printing is performed in a one-to-one model.

In the next essential step, the model was validated against the navigation system (*Kick—Brainlab*, Munich, Germany) in terms of its correspondence to the CT/MR image data. This can be done by region registration based on the acquisition of landmarks. Alternatively, registration using X-ray in two planes and subsequent matching for cross-sectional imaging is possible. The latter requires an additional contrast for X-rays, e.g., by means of zinc-containing coating. If there is sufficient accuracy within the error specified by the navigation manufacturer, it is possible for the surgical team to comprehensively evaluate the subsequent surgical steps and resection planes, as well as reconstruction options.

A systematic literature search was conducted via PubMed and Scopus plus Medical Subject Headings (MeSH) terms. After screening for duplicates, all titles and abstracts were reviewed. To maintain a broad perspective and include relevant articles, the references of the eligible articles were included in the search and added to the review where appropriate.

Articles that met the following exclusion criteria were not considered: studies on maxillofacial, cranium, or extremity tumors; studies in languages other than English or German; conference proceedings; studies solely for patient education; and studies solely on the printing of biomaterials.

## 3. Results

All surgeries were conducted as conventional operations, except the osteotomies and the speed burring, for which the navigation system was used. Image-to-patient registration was performed using one of the following three methods: paired points, surface matching or matching radiographs in two planes (*Xspot*, *Brainlab*, Munich, Germany).

### 3.1. Case 1

**Figure 2:**
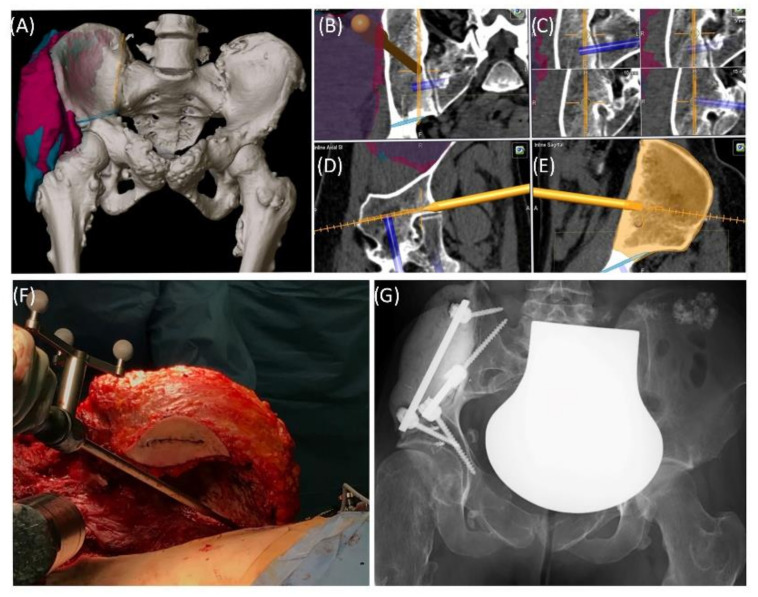
Case 1: Preoperative planning and segmentation of tumor volume components [bony (pink) and soft tissue (teal)] (**A**); resection planes [transverse (orange) and axial (blue)] during surgery with navigated chisel (orange) (**B**–**E**) intraoperative situs during resection (**F**); postoperative radiograph with reconstructed iliac defect (**G**).

Case 1 involved a 22-year-old female patient suffering from a multiple cartilaginous exostosis disease with a secondary peripheral chondrosarcoma G3 of the right ilium without metastasis. The resection was performed as navigated internal hemipelvectomy (P1a), followed by augmentation of the right ileum with a standard screw-rod system (*Expedium*, *DePuy Synthes Spine Inc.*, Raynham, MA, USA) and revision cement (COPAL^®^ G+C, Heraeus, Wehrheim, Germany) (Figure 2). This procedure allowed preservation of the hip joint. Trochanter resection was followed by reconstruction of the ventral hip joint capsule and reinsertion of the musculature using an attachment tube (*Implantcast*, Buxtehude, Germany). During treatment, superficial dry skin necrosis at the Enneking approach site occurred without signs of infection and eventually healed. The first follow-up 3 months after surgery showed a stable and increasingly fluid gait pattern with a positive Trendelenburg sign. The range of motion of the right hip joint was fully preserved. At the 15-month follow-up, the patient was free of pain and was able to walk a three-kilometer distance. The native X-ray and MRI of the pelvis showed no signs of tumor recurrence.

### 3.2. Case 2

**Figure 3:**
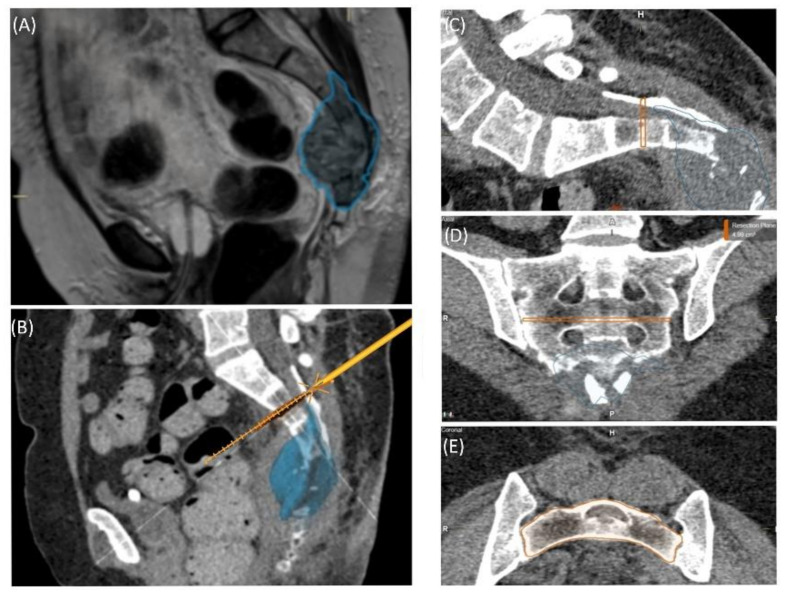
Case 2: Tumor segmentation (**A**) and resection plane planning (**B**), as well as preoperative validation of model accuracy with a navigated chisel resection of the 3D-printed model (**B**–**E**).

**Figure 4:**
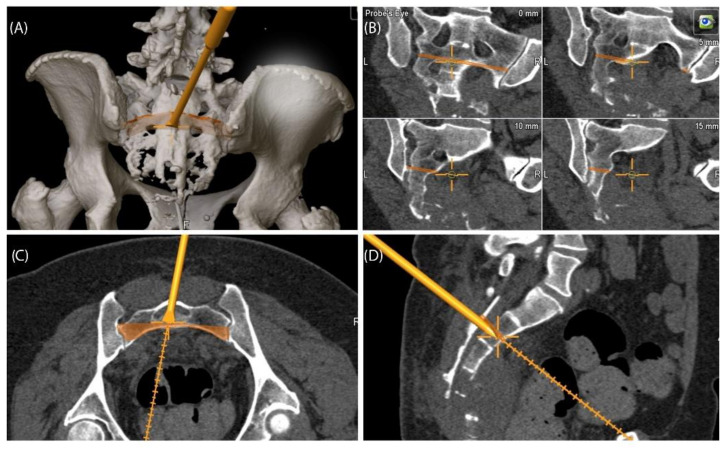
Case 2: 3D model of navigated chisel resection (**A**); resection planes in the axial and transversal planes (**C**,**D**); intraoperative view of the navigated chisel with crosshairs showing the location of the tip (**B**).

A 59-year-old female patient was diagnosed with bone metastasis of endometrial carcinoma of the sacrum with increasing and immobilizing pain. Primary radiation was discussed, but primary surgery and adjuvant radiation were preferred. Preoperatively, bladder/rectum dysfunction and a bladder-vaginal fistula were already present due to significant metastatic progression. The goal of 3D model-based planning and navigation was marginal resection of the metastasis in terms of a partial sacrectomy below S2 with ligation of the dural tube and the descending nerve roots below S2 (Figure 3 and Figure 4). Repeated revision surgery and systemic antibiotic treatment after wound infection were necessary. At the two-month follow-up, further metastases with pulmonary foci, which did not exist preoperatively, were found. Palliative treatment and radiation followed.

### 3.3. Case 3

**Figure 5:**
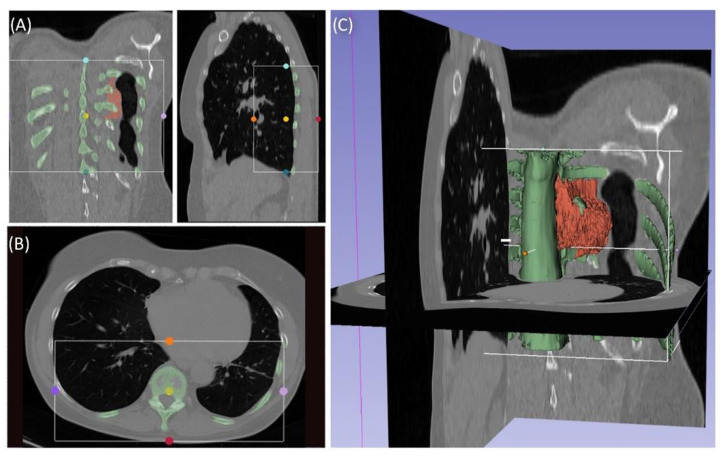
Segmentation of a thoracic chondrosarcoma and preparation for 3D model printing; cross-sectional segmentation (**A**,**B**) and 3D model with tumor volume (red) (**C**).

**Figure 6:**
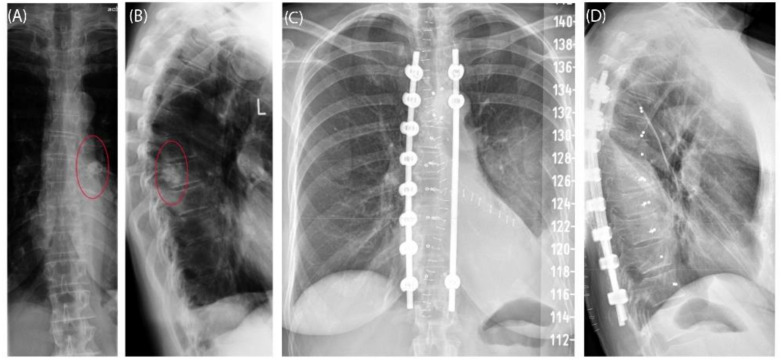
Case 3: Preoperative radiographs in frontal (**A**) and lateral (**B**) views, red circles indicate the bony tumor mass; postoperative radiographs in frontal (**C**) and lateral (**D**) views.

Case 3 involved a 63-year-old female patient with a diagnosed inner thoracic chondrosarcoma (G2) of the 9th left rib. The soft tissue component extended from the 7th to the 11th rib next to the aorta and lungs (Figure 5). After CT-MRI fusion and planning, marginal resection was possible by combining a partial corpectomy T7–T11 with laminectomy T7–T11 and partial resection of ribs 7–11 on the left side. Reconstruction was carried out by instrumentation spondylodesis with a screw-rod system from T5-T11 and extensive coverage with bovine pericardium (Baxter, Deerfield, IL USA) (Figure 6). The postoperative histopathological examinations confirmed tumor-free resection margins and revealed a high-grade tumor (G3). After adjuvant radiation and a four-month postoperative follow-up, there was no sign of local recurrence. The patient returned to daily life without any sensomotoric deficit.

### 3.4. Case 4

**Figure 7:**
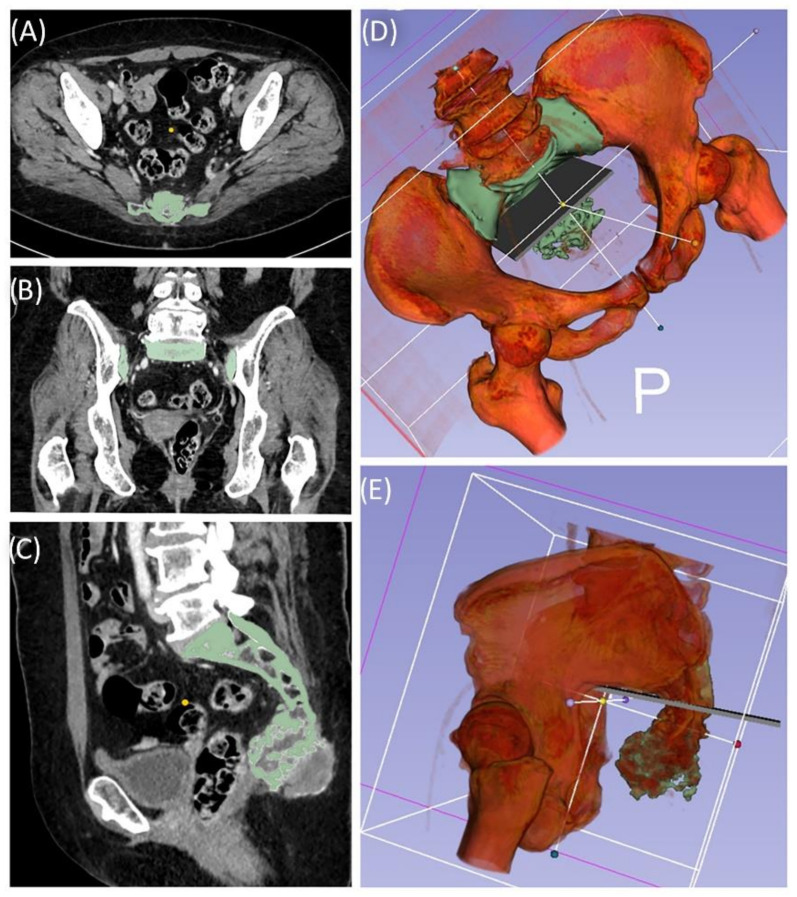
Case 4: Segmentation and resection plane planning of a high-grade osteoblastic osteosarcoma of the sacrum; cross-sectional segmentation (**A**–**C**) and 3D model with the resection plane as the volume (gray) (**D**,**E**).

A 49-year-old female patient was diagnosed with an unclear sacral/coccygeal mass that was painful on palpation. The patient reported load-dependent coccydynia for 6–7 months with no typical radiculopathy, intact sensorimotor function, and regular fecal/urinary continence. After biopsy, high-grade osteoblastic osteosarcoma was diagnosed, and four cycles of neoadjuvant chemotherapy were administered according to the EUROBOSS protocol. Navigated resection of the sacral region S2/S3 to S5, including the coccyx (Figure 7), reconstruction of the defect of the rectal intestinal wall and reconstruction using a Vicryl mesh loaded with gentamycin chains, were conducted. Histopathologically, a R0 resection with a regression degree of 4 according to Salzer–Kuntschik was confirmed. Incontinence remained due to the resection of the sacral nerve roots. Because of reduced physical status, chemotherapy was not continued postoperatively.

### 3.5. Case 5

**Figure 8:**
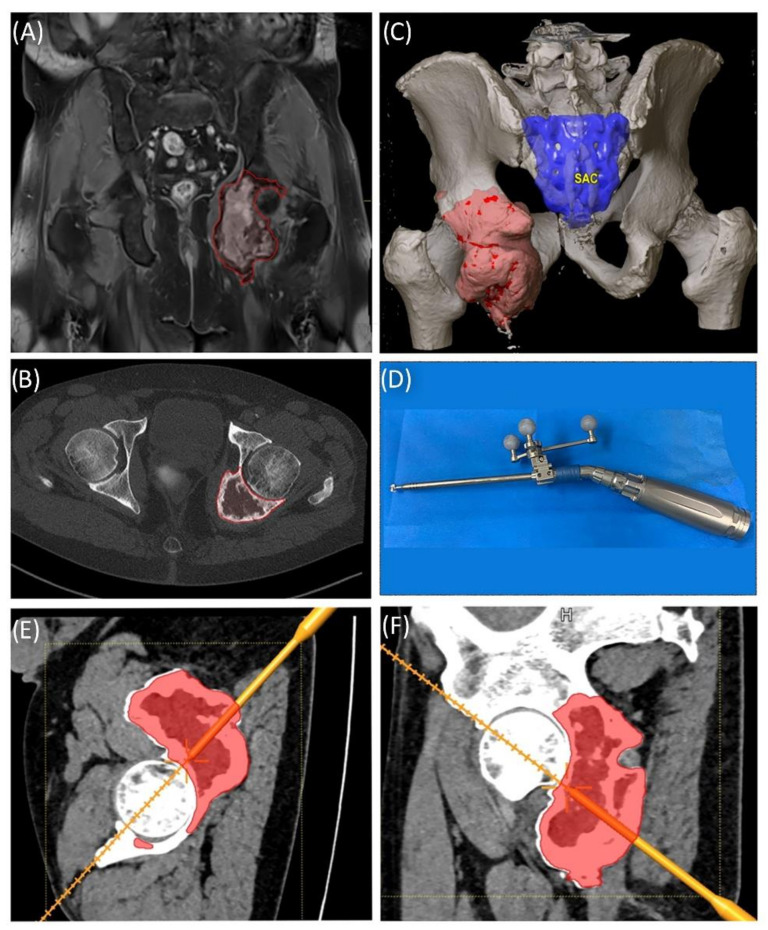
Case 5: Segmentation of the giant cell tumor in magnetic resonance imagining (MRI) (**A**) and computed tomography (CT) (**B**) scans, segmented tumor volume (**C**), high-speed burr navigation (**D**), intraoperative navigation of the tumor volume with the high-speed burr tool tip (**E**,**F**).

**Figure 9:**
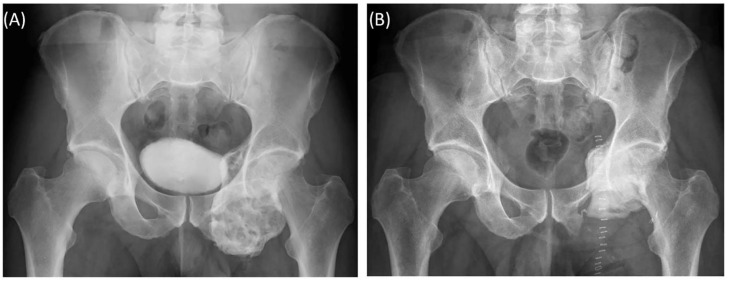
Case 5: Preoperative plain radiograph of the acetabular giant cell tumor (**A**) and postoperative result after resection of the dorsal parts and intralesional curettage with a navigated burr and augmentation with polymethyl methacrylate (**B**).

A 58-year-old male patient was diagnosed with a giant cell tumor of the left pelvis in 2013. After five years of conservative treatment with denosumab, he developed recurrent jawbone osteonecrosis. Therefore, the treatment was stopped in 2018. Consequently, tumor progression with increasing symptoms occurred, and surgical resection was indicated after rebiopsy and histopathological confirmation. Intralesional resection was performed via high-speed burr curettage followed by adjuvant polymethyl methacrylate filling (Figure 8 and Figure 9). This approach allowed preservation of the hip joint. Foot drop on the left side was noted postoperatively, and hip flexion was restricted to 60° for 4 weeks postoperatively to avoid luxation. At the 6-month follow-up, the patient had free range of motion of the hip in all directions except for a maximum of 10° external rotation. The foot drop increasingly improved, and orthosis was no longer necessary at this point.

### 3.6. Review of Literature

The initial search with “bone tumor AND 3D printing” resulted in 254 PubMed and 205 Scopus matches up to 27 October 2020. Of these, the majority were articles and case reports; some were reviews (21 in PubMed and 22 in Scopus), and only one was a systematic review.

**Figure 10:**
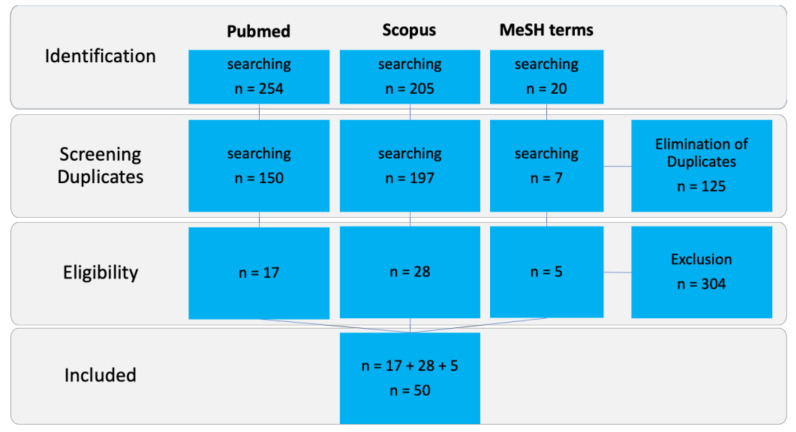
PRISMA (Preferred Reporting Items for Systematic Reviews and Meta-Analyses) flow chart with 50 articles included.

The results for “(printing, three-dimensional [MeSH Terms]) AND (surgery, computer-assisted [MeSH Terms]) AND (bone and bones)” included 138 articles but decreased to seven when excluding the terms “cranio”, “maxilla” and “facial” (Figure 10).

## 4. Discussion

In our case series, we combined navigated tumor resection with preoperative planning by 3D modeling and printing and successfully evaluated the navigation steps from the printed model for complex bone tumor resection of the trunk. From our process and clinical follow-up, we are encouraged and convinced of its safety. However, this study involves the development and testing of an experimental approach. The development of an in-house standard with further quality assessment is a future task. For this reason, a review of the current literature was conducted, and key aspects were identified that could help with in-house validation.

The overall 5-year survival of pelvic sarcomas is reported to be less than 28% [22]. Oncological and functional outcomes depend on several factors. One factor is a tumor-free margin which can compete with the wish of a good functional outcome. Without any technical assistance, e.g., navigation, even an experienced surgeon is challenged to consequently achieve a clearly defined margin with the complex anatomy of the pelvis [23]. Clinical findings of conventional resections report an incidence of intralesional resection at the pelvis of 29% and a recurrence rate of 27% [24]. Therefore, the request for improvement is justified.

In a case series of 13 patients (seven with pelvic tumors, six with upper extremity tumors), individual implants for reconstruction were presented by Angelini et al. in 2019. At the two-year follow-up, at least good functional results were reported, with a complication rate of 38.5%. The authors suggested an experience-based decision-making algorithm for deciding on reconstruction of the pelvis rather than modular prosthesis for individual implant construction depending on the resection level [25]. No information on the time span for planning, construction and manufacturing until surgery was given.

The indirect effects of 3D printing on surgical procedures, including reduced surgery time, reduced blood loss or radiation time, are reported in the literature [26,27]. This may contribute to a reduced complication rate of wound infections and wound healing disorders [6,7,8] and therefore enable earlier adjuvant therapy initiation.

As another result of these observations, it is clear that 3D printing in the context of preoperative planning helps to avoid wasting resources, e.g., by not having to try out appropriate instruments in the operating room first, thus avoiding unnecessary reserialization or disposal [28].

As early as 2018, the 3D printing Special Interest Group (SIG) unveiled a rating system that highlighted the suitability of medical 3D printing for clinical use, research, scientific, and informative purposes [28]. According to this evaluation system, four particularly suitable main groups were identified: craniomaxillofacial trauma, congenital malformations, acquired/developmental deformities, and neoplasms. In particular, the last group applies to the cases presented here. While Chepelev et al. mainly focused on technical aspects, from a tumor orthopedic point of view, there are additional aspects to be considered regarding disease progression, therapy stages, and surgical safety.

In the course of this review, we would like to take a closer look at further aspects and perspectives of recent findings from research.

### 4.1. 3D Printing for the Treatment of Tumors

Future trends in the treatment of bone metastasis aim to improve local recurrence control and to overcome systemic side effects by local solutions using therapeutic osteoinductive and osteoconductive adjuvants in 3D-printed biodegradable spacers with structural support. In their review on advances in personalized metastatic therapy, Ahangar et al. mentioned their promising results from an in vitro study on drug-eluting nanoporous 3D-printed scaffolds [29]. They concluded that tissue-engineered bone substitutes have high potential to circumvent the limitations of conventional therapy strategies, e.g., donor site morbidity of bone grafts. Despite these encouraging innovations, clinical data and experience are still limited.

Haleem et al. 2020 conducted a literature review on the basic applications of 3D printing to improve tumor therapy. In their article, the authors grouped the applications of 3D printing as follows: replicas of cancerous parts, 3D phantoms of tumors and organs, treatment of tumor tissue, study cancer growth, monitoring of cancer treatment, teaching tools, etc. With their concise look at 3D printing on bioprintables, they shed light on the potential of this manufacturing technology for cancer therapy. However, this has to be distinguished from classical AM methods using plastics, ceramics, or metals due to the effects on active cells and metabolic activity.

Consequently, there is no reference in their work to an application using or combining 3D printing with intraoperative navigation or custom endoprosthetics [30].

### 4.2. Patient-Specific Devices—Personal Surgical Instruments (PSIs)

Patient-specific devices are increasingly used and include any anatomy-fitted jigs, cutting guides, templates for implant positioning and implants [12,13,14,27,31,32,33,34]. Numerous case reports illustrate and complement the versatility of using personal surgical instruments (PSIs) [35].

Although software and development are steadily improving, technical demands for accurate automatic algorithms for medical image processing, especially in the field of image segmentation and registration, are unabated. The user friendliness of powerful software tools is only one factor, among others, to provide access to a patient-specific design of instruments and implants [36]. Aside from the technical aspects, the use of these individual solutions also raises several questions in clinical practice.

The use of custom instruments has raised the question of possible effects on wound healing. For pelvic tumor surgery, complications such as wound infections can occur, e.g., after a longer operation time and extended resection, including custom implants [37]. In a retrospective analysis, Shea et al. presented a large cohort of more than 100 patients who received patient-specific devices or intraoperatively used anatomical models within a 4-year period. In their study, they presented a detailed workflow and examined the infection rate associated with the intraoperative patient-specific models used after hydrogen peroxide plasma sterilization. In the published results, the infection rate of 7% was not significantly different from that with standard surgical procedures. Shea et al. concluded that their process is safe for continuation and implementation elsewhere [38].

Another significant aspect from the orthopedic tumor point of view is the recurrence rate. In a prospective study of nine cases in comparison to a historical control group of 19 cases, Robin et al. reported a reduction in the local recurrence rate to zero with the use of 3D-printed patient-specific instruments [39].

The use of customized 3D-printed implants has introduced a wide range of possibilities [40]. However, outcome and function depend largely on correct positioning and the quality of the implant-bone interface in terms of primary and secondary stability [33,41]. In particular, the design of porous implant structures has an important influence on osteointegration, osteoconduction and osteoinduction and should match the patients’ needs [42,43]. The choice of optimal design parameters is only one of many factors that still require close collaboration between clinicians and engineers. The articles by Kwok et al. 2016 [33] or Mitsouras et al. 2015 already illustrated this clearly [44].

Since the cutting templates and individual implants, or at least the manufacturing process itself, require approval as medical devices, the application may be limited to a small circle of users; in our experience, this type of approach cannot feasibly be made available to all users in a timely manner at present.

### 4.3. Navigation-Assisted Surgery

Cho et al. investigated the long-term outcomes of navigation-assisted bone tumor surgery in 18 cases and used software-based preoperative resection planning. The described MRI to CT image-to-image registration was performed using K-wire or resorbable pin placement before imaging. The author’s defined acceptable error was <2 mm. Comparison of the histopathological margins to the preoperative plan showed a maximum error of 3 mm. The time for navigation setup was reported to be less than 30 min [9]. The average intraoperative navigation time during surgery was reported by Wong et al. to be less than 25 min [21].

In another retrospective analysis of pelvic and sacral primary tumors of navigation-assisted resections, Jeys et al. demonstrated that navigation helped to preserve sacral nerve roots, convert inoperable findings into operable ones, and avoid hindquarter amputations. The authors concluded that the risk of intralesional resection and, at least in an over short observation period, the local recurrence rate can be positively influenced by navigation [24].

A direct comparison of standard versus navigation-assisted resection was performed by Laitinen et al. in a retrospective study. They confirmed the aforementioned findings and additionally reported significantly reduced blood loss and a non-significantly reduced operation time. Based on these results and with reference to other authors, the authors concluded that navigation allows accurate visualization of the tumor, predictable alignment of osteotomies, and accurate placement of custom prostheses. Accordingly, more accurate resection may allow preservation of articular surfaces, which is unlikely without navigation [5].

Consistently, the authors concluded that computer-assisted navigation reduces the risk of intralesional resections and recurrence rates and furthermore improves functional outcomes.

Image-to-patient registration errors less than 2 mm seem to be a minimum requirement [9], but less than 1 mm is considered acceptable [5,24,45].

For spinal screw insertion, a surface registration accuracy of 0.5 mm is advised in CT-guided navigation as the clinical threshold [46]. Thus, CT scan data should provide similar accuracy, depending on the anatomic region and surgical needs, with commonly reported slice thicknesses between 0.5 for fine bone structures and 2.0 mm for larger structures, such as the pelvis and sacrum [47,48]. In line with these requirements, our scan parameters ranged from 0.6–1.5 mm. The detailed scan parameters are presented in Table A1 in Appendix A.

Cartiaux et al. demonstrated improved accuracy with navigation in simulated bone tumor surgery compared to freehand resection and concluded a possible benefit for pelvic tumor surgery by achieving clinically acceptable margins [49].

### 4.4. Navigation and Anatomic Modeling

The use of 3D anatomical models in combination with intraoperative navigation for pelvic tumors has been reported in the past. Zhang et al. described a case of hemipelvectomy and concluded that the combination allows a more targeted approach and safe osteotomies. However, in their study, the 3D model based on CT data was used only for spatial orientation. Navigation of the model for preoperative preparation did not occur. Accordingly, it is not clear whether a control of the dimensions and thus an actual 1:1 model was used [50].

Another case report by Heunis et al. describes a similar approach. The model was created by an external company, and intraoperative navigation was performed, in which the model only served as a visual guide and supplementary physical orientation aid [51].

Considering these rare literature results, our case series represents the first comprehensively described combination of preoperative planning and navigation on individual 3D models.

### 4.5. Accuracy of PSIs and Individual Implants vs. Navigation

The resection error ranges from 1 to 4 mm from planned resection to implant position with PSIs [52].

In a subsequent study, Cartiaux et al. compared the osteotomy results from three different modalities with their pelvic bone model: free hand, PSI and navigation assisted. They evaluated the location accuracy of defined osteotomy planes at the pelvis with a PSI in a saw bone model with the aim of a 10 mm safe margin to a virtual tumor volume. Compared to free-hand osteotomy, this approach yielded a significantly higher average accuracy of 1.0 to 3.7 mm and a lower difference of 3.1 to 5.1 mm from the targeted safe margin. The navigation-assisted osteotomy results were comparable, suggesting an equivalence to the PSI. In this model, the PSI was superior to navigation in terms of time consumption, which they explained by the predefined best-fit position of the PSI on the bony surface [53]. While these results are from an ideal in vitro setup without soft tissue and vulnerable in situ structures, the reported time consumption for the setup of the navigation system and patient registration are routine and system dependent.

In a prospective cohort of 11 patients, Gouin et al. resected pelvic bone tumors using PSIs. The authors defined different distances (from 3 to 15 mm) as desired safe margins. The location accuracy of the PSIs was reported to range from 1.5 to 4.4 mm. Deviations from the desired margins were within the acceptable range. One instance of local recurrence after 18 months of follow-up was reported. These results are comparable to those of a cadaver study from Sallent et al., which showed that a mean improvement of the accuracy up to 9.6 mm in sacroiliac osteotomies could be achieved by using PSIs [54]. The authors concluded that PSI-assisted surgeries can be performed safely with an accuracy that is clinically relevant for pelvic bone tumor surgery. The limitations of the study included the lack of a control group and randomization. Nevertheless, this study shows that multiplanar resection of tumors benefits from comprehensive preoperative planning and that the presented approach has the potential to improve patient safety and thus recurrence-free survival under certain conditions.

Depending on the rapid prototyping technology, PSIs are provided with a dimensional tolerance, e.g., 0.2 mm. These instruments are often made of plastics and carry a risk of unintentional material removal during drilling or osteotomy. The possible unintentional retention of the fine particles in situ must be viewed extremely critically.

One possible critical aspect of individual surgical instruments and implants is the time-consuming design and verification process, including biomechanical considerations. Even in a print-on-demand setup with professional engineer support in a standardized process, this may take several months, excluding analysis with the finite element method (FEM) [52,55].

### 4.6. Biomechanical Considerations

The FEM supports the mathematical consideration of complex relationships of material properties, such as stresses, reactions in the implant bearing with bearing forces and deformations. The models are often validated empirically. As early as the 1990s, Dalstra and Huiskes used the FEM to investigate the main loading zones of the pelvis. With their results, they were able to show that despite considerable variance in the force applied to the hip joint, the resulting vector affects the anterior and superior portions of the acetabulum. Accordingly, the most important load-bearing portions of the pelvic bone are the sacroiliac joint and pubic symphysis, with load transfer through the superior acetabular rim, the incisura ischiadicae, and, to a lesser extent, the pubic bone [56].

The importance of the biomechanics of the pelvis and hip joints was revealed by several preliminary works and different parameters [57,58]. Lee et al. showed with an FEM investigation of a case of periacetabular osteotomy that by changing the joint angles by a few degrees, the contact area of the joint partners could be increased by a factor of 10, and surface pressure in the joint could be halved [57].

Simulation using an FEM approach can help to make the decision between several reconstruction and anchorage alternatives [52,59,60]. The anatomical variability of different individuals is difficult to account for in models based on image data of one patient. Sophisticated models are often based on extensive validation with numerous material parameters. These are typically limited to passive structures such as bone, cartilage, and ligaments [59]. Consideration of active muscle forces is the subject of current research, and to date, metabolic processes for prognosis can hardly be represented in an individual model.

For a realistic biomechanical assessment, surgical planning and, if necessary, an individual endoprosthesis, the open source database OrtohLoad.com (accessed on 28 October 2020) offers additional comprehensive in vivo data samples of joint contact forces and moments, whole body kinematics and ground reaction forces [61].

This existing knowledge should be considered when planning resection levels and individual reconstructions, and recent developments in simulation should be carefully implemented and included in considerations when possible. This can help to identify unfavorable loading situations of implants to optimize implant positioning and thus effectively reduce risks such as loosening or material failure.

Beyond that, however, in a clinical setting, it is questionable whether the time required for an individual FEM approach justifies planning for tumor resection and reconstruction.

### 4.7. Regulatory and Technical Considerations

As already stated by Chepelev et al., “The United States Food and Drug Administration (FDA) classifies medical 3D printing software into design manipulation software that enables medical device design and modification and build preparation software that enables the conversion of the digital design into a file format that is 3D printable […]” [62]. The FDA expert group identified various critical aspects in the AM process. In their non-binding recommendation, they describe these considerations, e.g., the effects of medical imaging: quality and resolution for matching, smoothing or image processing algorithms that could influence the dimensions compared to the referenced anatomy, effects from soft and bony tissue structures and uniqueness of anatomic landmarks. As an example, the geometry and volume of a tumor could not be consistent over time or may have been influenced by patient movement during examination, leading to a mismatch of imaging and patient features. Therefore, a risk-based approach for process validation is recommended to prevent these effects/meet these constraints. This also includes but is not limited to a validation of [63]:

1. File format conversions, for example, when changing the applied software.
2. Digital device design in a general four-step process: 1. The placement of the model in the build volume, 2. the addition of support structures, 3. slicing and 4. build path generation. For example, support structures can influence surface quality or geometry, or warping can occur.
3. Machine parameters and environment: preventive maintenance intervals, calibration, environmental conditions in the build volume (temperature, atmospheric composition) and adjustable machine parameters should be documented.
4. Material controls: the raw material should be provided with detailed data sheets by the manufacturer. The printing process can influence these parameters. Therefore, further tests may be required for the processed material.
5. Postprocessing: this may include, among other things, the finishing of surfaces to improve surface quality. However, the device performance must not be negatively affected and must be ensured by suitable methods, such as mechanical tests.
6. Process validation: a manufactured model or device must have clear and verifiable quality characteristics. The variability of input parameters and manufacturing steps has an influence on this. If the AM process result is not fully verified, process validation must be performed. Test coupons that are printed together with the model or device can help with process validation. These have defined geometries and surface structures and can be positioned appropriately to serve as a worst-case scenario and assure quality control for the actual AM print job.

However, there is often a lack of in-house expertise on implant design and the necessary on-site manufacturing technology with a corresponding comprehensive quality management system for the design and manufacture of medical devices, e.g., according to ISO 13485 [55].

Even if the result of the in-house process is an anatomical model and not a higher classified patient-specific device, compliance with regulatory requirements is mandatory. The European Union (EU) Medical Device Regulation (MDR) guidelines require that all processes for the design and manufacturing of a device for medical use be registered. A certain imprecision of the MDR guidelines remains with regard to segmentation of image data and software packages used. It should therefore be in the interest of every facility to comply as closely as possible with the MDR guidelines for anatomic models. Processes described in detail by various authors, such as Chepelev et al. 2018 and Willemsen et al. 2019, who have already worked out a procedural workflow in consideration of the regulatory framework, could serve as a blueprint [28,55].

### 4.8. Costs, Print Time and Quality

In the literature, 3D printing has been repeatedly evaluated for its effects on surgical procedures. Reduced surgery time and reduced blood loss are among them [27]. However, there is also a comprehensive financial analysis of operating room costs. In a review by Ballard et al., the repeatedly observed reduced surgery time of approximately 20–60 min on average indicates a significant cost reduction of the equivalent of 1200–3200 euros in the operating room. However, this is counterbalanced by the resources and expertise required for 3D printing. According to this, a break-even point is already reached at 1.2 models/week [64].

The choice of the printing method determines the extent of the investment and operating costs for an on-site printing system in a hospital. Fused filament fabrication (FFF)/fused deposition modeling (FDM) filament printers have become accessible for lower four-digit amounts (in euros), while PolyJet (liquid photopolymer jet) printers and other processes, such as laser sintering (SLS) or stereolithography (SLA), easily cost high six-digit amounts. In addition to the initial purchase expense of a device, higher costs can quickly be incurred for maintenance and materials. It is undisputed that filament printing is currently the most cost-effective method. Beyond this, the choice for the printing method should also depend on the intended use of the printed result (anatomic model, PSI or PSI) [44].

Chen et al. tested different printing technologies with different settings, such as infill, layer height, and orientation on a model print bed, and assessed their effect on the estimated duration and cost of anatomical models. The FDM method proved to be the most cost-effective method, with a cost of less than 20 USD per printed model. While 5% changes in infill had no significant impact, different orientations in the print bed resulted in cost increases of 35% and print duration increases of 40%. While printing with other methods is significantly more cost-effective, the expected printing time is up to ¾ shorter with the PolyJet method. However, the actual time required for a print job can differ significantly from the estimate [65]. With their study, Chen et al. provided a comparison of different AM technologies and also considered the orientation of the print model. However, technology is developing rapidly in a fast-growing market, and their overview is a limited snapshot. Each technology has its benefits, and there is an increasing number of options for materials or even compounds in the field of AM. Therefore, the choice for the right printer should never be influenced solely by the purchase price.

The cost and effort of postprocessing or maintenance have not been specified in the literature as of the time of our review but should be considered when choosing the printing method and printer.

Due to the high resolution of the printers, the achievable quality of the print result usually exceeds the quality of the diagnostic imaging used for modeling, since slice thicknesses of several millimeters are often used here. However, numerous process variables must be considered for the print result.

Beyond this, print time and accuracy are dependent on essential parameters such as the resolution. There is a difference in printing resolution in the xy-direction, which represents the horizontal plane, and the z-resolution, which is given with the layer height of the print [66]. In the field of FFF/FDM printers, the normal resolution of the nozzle is 0.4 mm. The laser resolution of SLS Printers is normally 0.1 mm, and the resolution of PolyJet printers is approximately 600 PPI. You can easily change the nozzle of an FFF/FDM printer to 0.6 mm or 0.8 mm. In contrast to the layer height, a software setting, these are hardware settings that can only be modified by exchanging printer components. It should also be noted that not every manufacturer offers this option.

**Figure 11:**
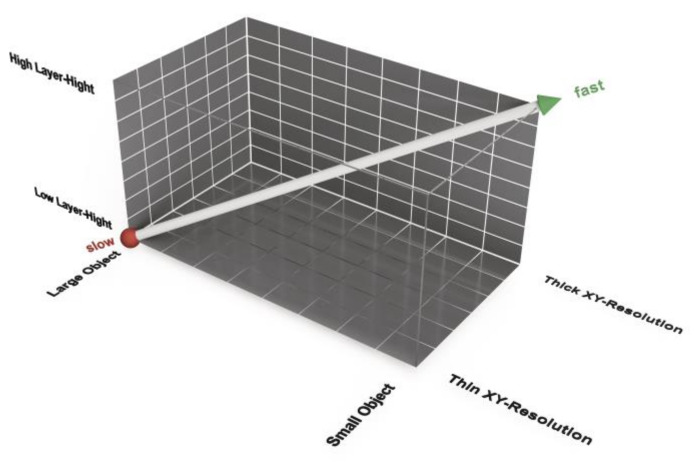
Print duration as a function of layer height, object size and xy-plane resolution for fused filament fabrication/fused deposition modeling (FFF/FDM) filament printing methods.

Consequently, the print-speed-accuracy matrix is not a 2D but a 3D function of layer height, object size and xy-resolution (Figure 11). A large object printed with a thin xy-resolution and a small layer height takes the longest print time. A small object printed with a thick xy-resolution and a large layer height can be printed faster. The accuracy correlates negatively with the print time. Furthermore, a large layer height may result in the loss of details in the z-direction. A higher xy-resolution allows a smaller radius at edges in the horizontal plane. This effect is comparable to industrial milling, where a small radius is the effect of a cutter with a small diameter.

### 4.9. Special Considerations and Pitfalls

- Input image quality, slice thickness/step phenomenon, kernel [67], noise reduction, mesh quality and number of triangles [44] scaling errors in import/export processes and wrapping.
- Smoothing of relevant structures.
- Resection margins should not be reduced.
- Rapid tumor progression must be considered, so images must be up to date.

When considering the aforementioned aspects and perspectives on accuracy, time savings, and additional costs for the possible applications of AM, regulatory aspects also play a key role. Finally, the production processes and the large number of selectable input variables are determining factors for the printing result. Therefore, statements on comparisons in the literature that suggest general validity should be interpreted with caution. For an illustrative model for discussion with the patient or for teaching purposes, different requirements must certainly be met compared to a model that is directly associated with the treatment of the patient, especially if it is used for the design of patient-specific templates, implants, or the planning of planes for navigated resection. For clinicians aiming to design their first experimental models, a step-by-step guide with valuable hints would be helpful to get started in this exciting but also partially complex field of medical 3D printing [62,68].

### 4.10. Advantages of Our Approach

The presented procedure allows a clinical team of surgeons and radiologists to become familiar with the techniques and possibilities of AM technologies. The generation of a 3D model and validation of its accuracy were the first steps towards patient-centered treatment using 3D printing in our hospital. The experience gained enables the development of a process standard in one’s own clinic, which can facilitate the step towards the development of individualized patient instruments and implants.

The benefits of our approach are as follows:

1. Replicates individual anatomy for preoperative planning, respecting critical structures.
2. Decreased surgical time and blood loss.
3. Preoperatively and intraoperatively adaptable with real-time visualization of multiplanar resections.
4. Variable navigated instruments (chisel, saw) for precise real-time visual control during resection.
5. Safe usability in simple and complex soft tissue situations.
6. Improved possibility for implant placement (without additional traumatic surgical preparation for drill holes or fixation pins for jigs and patient-specific instruments).
7. Decreased radiation exposure.
8. Supports tumor clear margin resection.

## 5. Conclusions

In our study, we presented a series of cases with complex bone tumor resection and described the combination of navigation and additively manufactured 3D models as an option for improved tumor resection planning.

The review of the current literature confirms the trend and diverse applications of 3D printing in the medical field. It also revealed that 3D printing in hospitals allows it to be used in a variety of applications and that processing is not standardized. Often, quality assurance standards are missing, partially because different perspectives and disciplines as well as regulatory aspects must be considered. For a safe procedure, the process should be transparently validated for patient-specific care at each hospital. For this purpose, it is recommended to have a clear process evaluation plan.

Like other authors, we are convinced that the increasing number of cases and presented evidence indicate that these methods can influence parameters in the treatment of complex musculoskeletal diseases. This includes patient and physician satisfaction, operation time, blood loss and various direct and indirect costs associated with shared decision making and patient-centered processes [28].

Three-dimensional (3D) printing has an increasing impact on precision medicine, and the presented case series represents a good alternative contributing to patient-centered individual care. It is expected that the future development of 3D printing will offer more solutions for patient-specific care in hospitals. It is our responsibility to take the next step and set up a framework in conformity with regulatory demands and to validate our approach to provide safety information and meet the highest medical standards.

## Appendix A

**Table A1:** Computed tomography (CT) scan parameters in cases 1–5, all in a matrix of 512 × 512.

Case	Device	Voltage (kV)	Electric Current (mAs)	Dose-Length Product (mGy*cm)	Slice Thickness (mm)	Pitch	Kernel
1	Siemens Somatom Definition Flash	100	2976	435	1.5	0.6	I26f
2	Siemens Somatom Definition Flash	120	1207	286	0.6	0.8	B20f
3	Siemens Somatom Definition Force	100	1145	233	0.75	0.8	Br69
4	Siemens Perspective	n/a	n/a	n/a	1.0	1.5	I80s
5	Siemens Somatom Definition Navigator S16	100	3862	313	1.0	0.9	B70F
